# Neoadjuvant Alectinib in a Patient With Anaplastic Lymphoma Kinase (ALK)-Mutant Stage III Lung Adenocarcinoma: A Case Report

**DOI:** 10.7759/cureus.95381

**Published:** 2025-10-25

**Authors:** Muzammil L Dastagir, Juhaina Bajaman, Jackson A Reynolds

**Affiliations:** 1 Internal Medicine, University of Alabama at Birmingham (UAB) Montgomery Campus, Montgomery, USA; 2 Hematology and Medical Oncology, Montgomery Cancer Center, Montgomery, USA

**Keywords:** alectinib, alk-positive, chemotherapy response, crizotinib, metastatic non-small cell lung cancer, non-small cell lung carcinoma (nsclc)

## Abstract

Lung adenocarcinoma is a heterogeneous malignancy requiring personalized therapeutic strategies. This case report details the management of a 33-year-old male never-smoker diagnosed with stage III lung adenocarcinoma harboring an Echinoderm microtubule-associated protein-like 4 (EML4)-anaplastic lymphoma kinase (ALK) fusion gene. Traditional chemoimmunotherapy has shown modest efficacy for this aggressive cancer subtype.

The patient presented with a large left upper lobe lung mass and mediastinal lymphadenopathy. Molecular testing revealed an EML4-ALK rearrangement and high programmed cell death-ligand 1 (PD-L1) expression. Initial treatment with chemoimmunotherapy was complicated by hypersensitivity reactions to nivolumab and paclitaxel, leading to a brief hospitalization. The treatment strategy was shifted to neoadjuvant alectinib, 600 mg twice daily. The patient tolerated the therapy well, experiencing only a transient photosensitivity rash. Subsequent imaging showed significant tumor reduction and no disease progression. In April 2024, the patient underwent successful surgical resection, revealing a complete pathological response with no residual tumor. This case demonstrates the efficacy of neoadjuvant alectinib in managing ALK-mutant stage III lung adenocarcinoma, highlighting the potential benefits of targeted therapy in the neoadjuvant setting. Further studies are needed to establish optimal treatment protocols for patients with ALK-positive lung cancer.

## Introduction

Non-small cell lung cancer (NSCLC) is the most common type of lung cancer, accounting for approximately 85% to 90% of all lung cancer cases, according to data from the Surveillance, Epidemiology, and End Results (SEER) Program. The advent of targeted therapies has significantly improved the prognosis for patients with specific molecular drivers, such as ALK rearrangements [1]. We present the case of a previously healthy 33-year-old male who developed cough and fatigue in December 2022, ultimately diagnosed with ALK-mutant NSCLC. Initial imaging showed a large left upper lobe mass with small mediastinal lymphadenopathy, and biopsy results confirmed poorly differentiated malignancy with thyroid transcription factor 1 (TTF-1) positivity. Subsequent molecular testing revealed an EML4-ALK gene rearrangement, which is seen in 3-7% of NSCLC cases and is increasingly targeted with ALK inhibitors [1].

Given the patient’s inability to tolerate traditional neoadjuvant chemoimmunotherapy due to hypersensitivity reactions, a targeted therapy approach with alectinib, an ALK inhibitor, was initiated. The patient exhibited an impressive clinical response with no evidence of disease progression following treatment. This case highlights the potential efficacy of neoadjuvant alectinib in patients with ALK-positive stage III NSCLC, offering an alternative to traditional therapies, particularly when they are not well-tolerated [2]. This report underscores the importance of personalized treatment strategies in managing advanced NSCLC and the promising role of targeted therapies in improving patient outcomes [2,3].

## Case presentation

**Figure 1:**
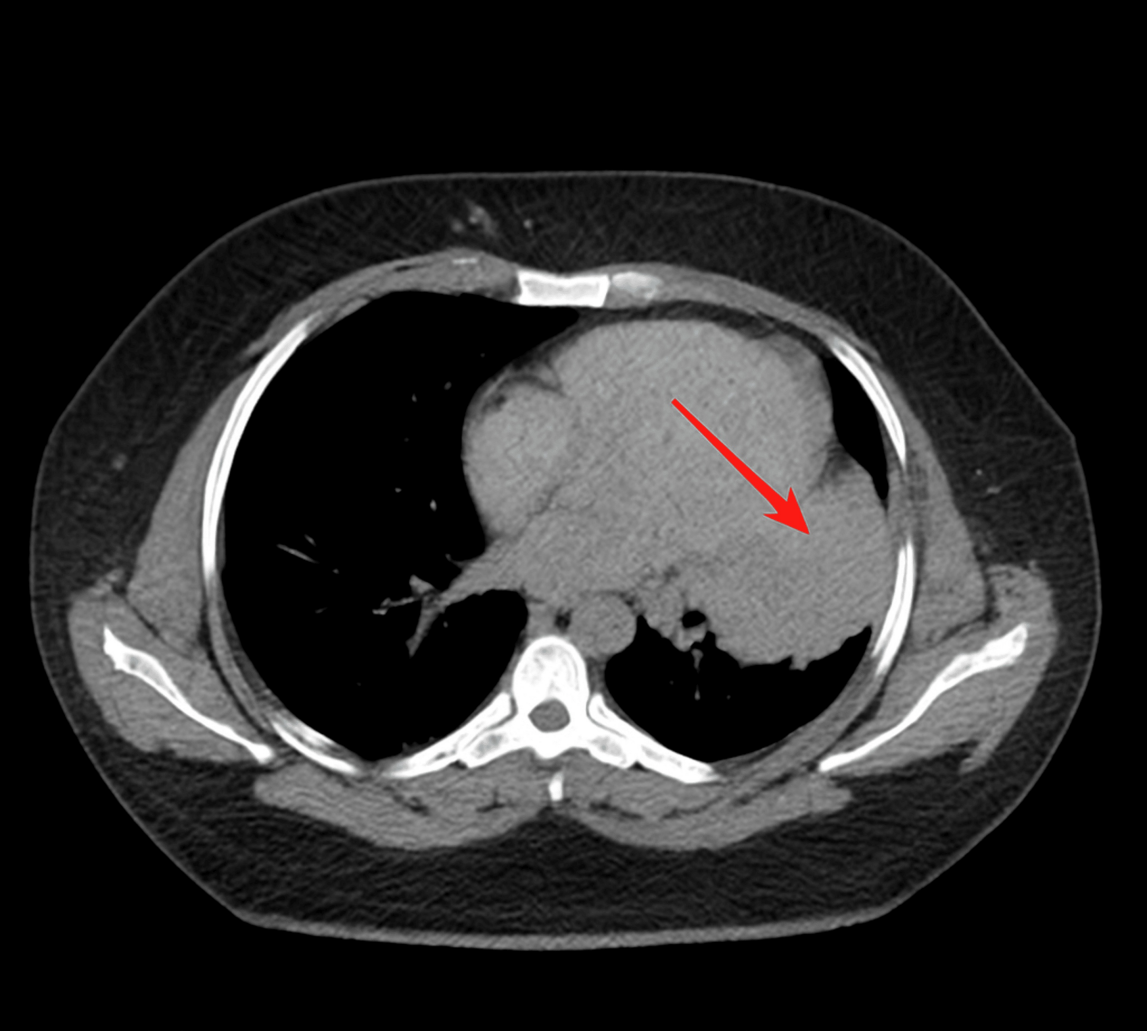
CT chest performed in March 2023 The image shows a left upper lobe (LUL) mass (red arrow).

**Figure 2:**
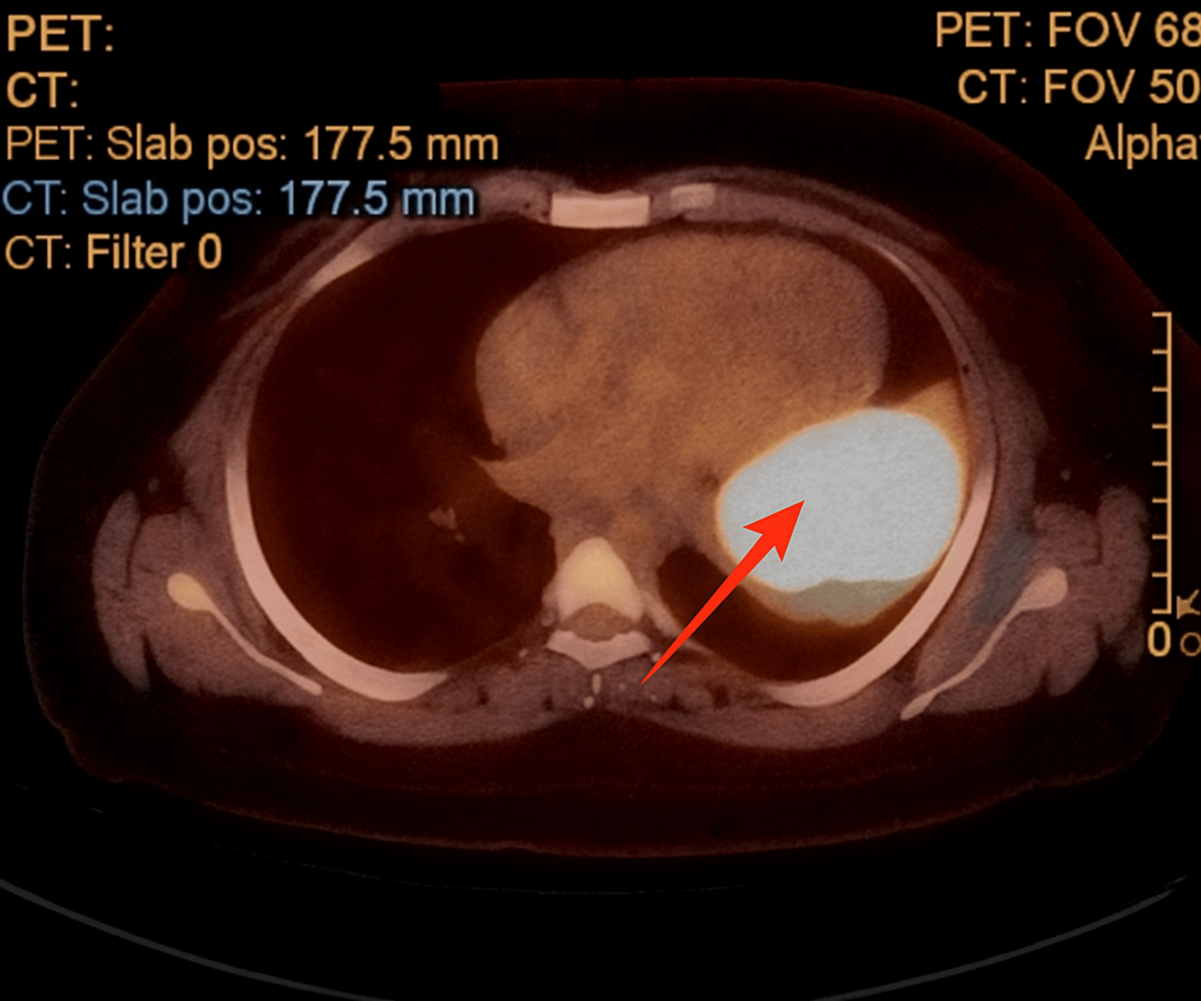
PET/CT scan image performed in April 2023 The image shows a hypermetabolic left upper lobe mass (red arrow). PET/CT: Positron Emission Tomography/Computed Tomography.

**Figure 3:**
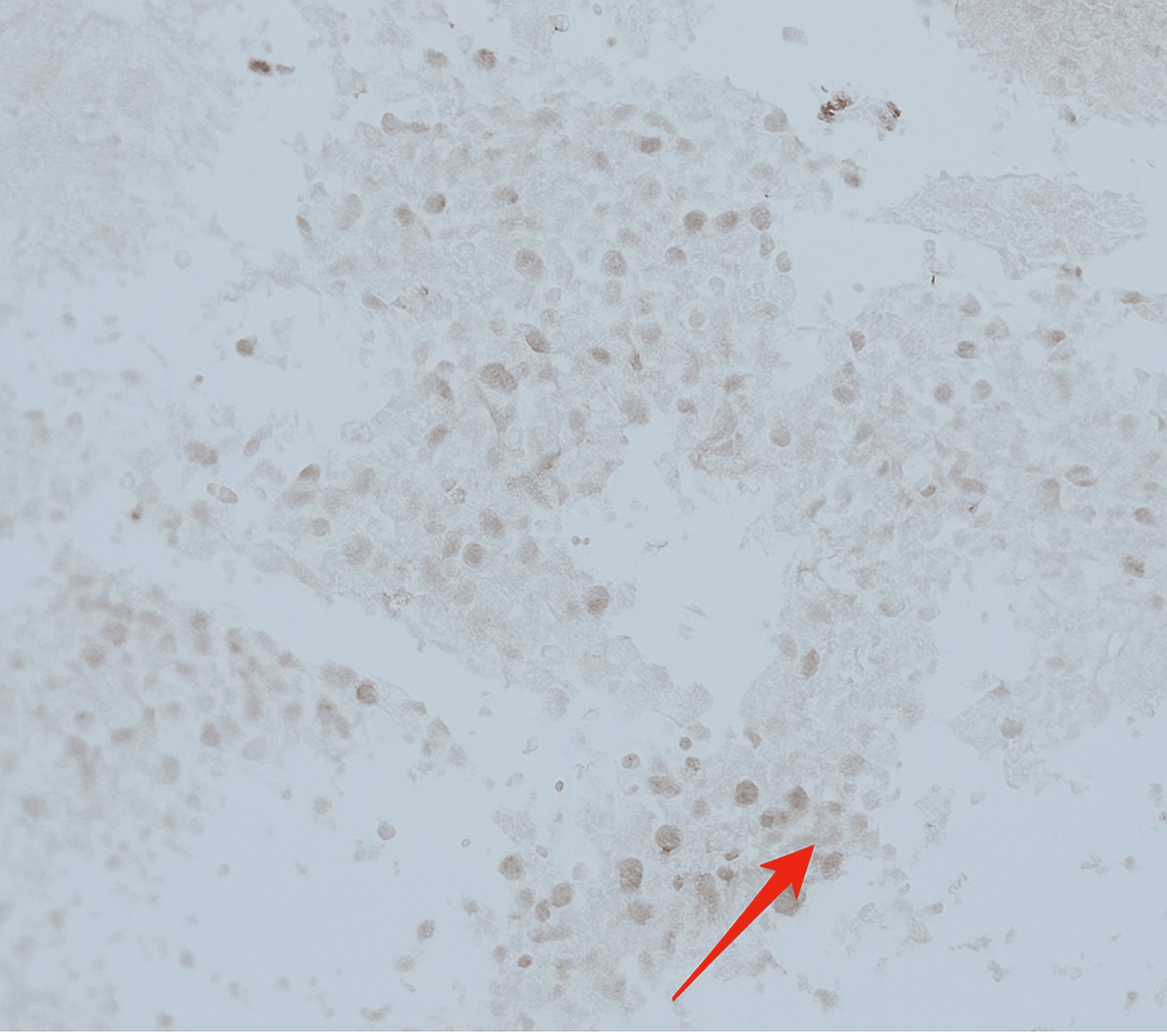
TTF-1 immunohistochemistry result The image shows thyroid transcription factor 1 (TTF-1) positivity (red arrow).

**Figure 4:**
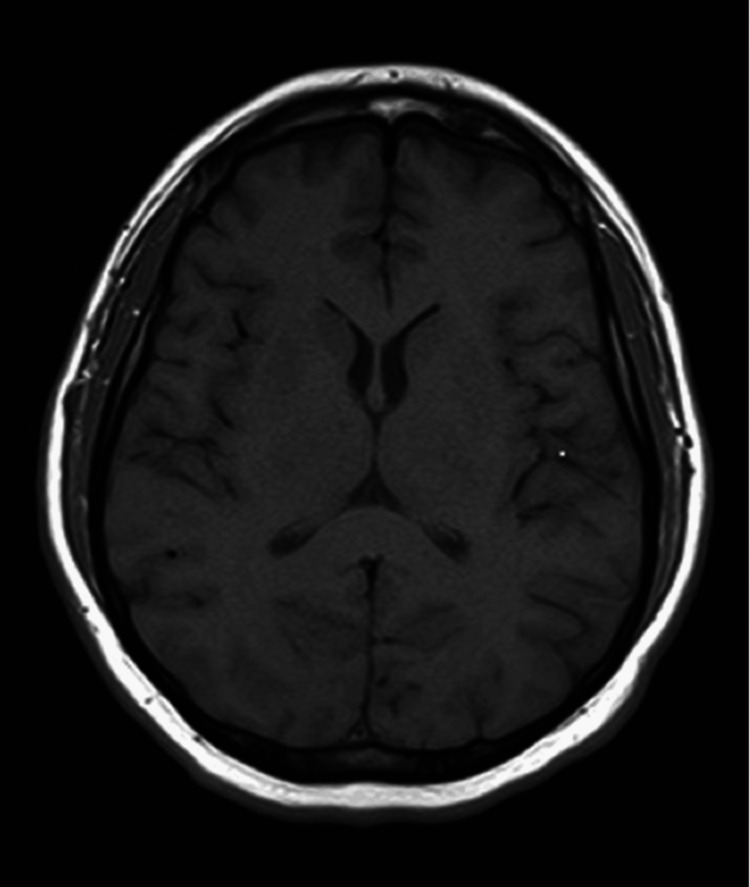
MRI of the brain showing no evidence of metastases

**Figure 5:**
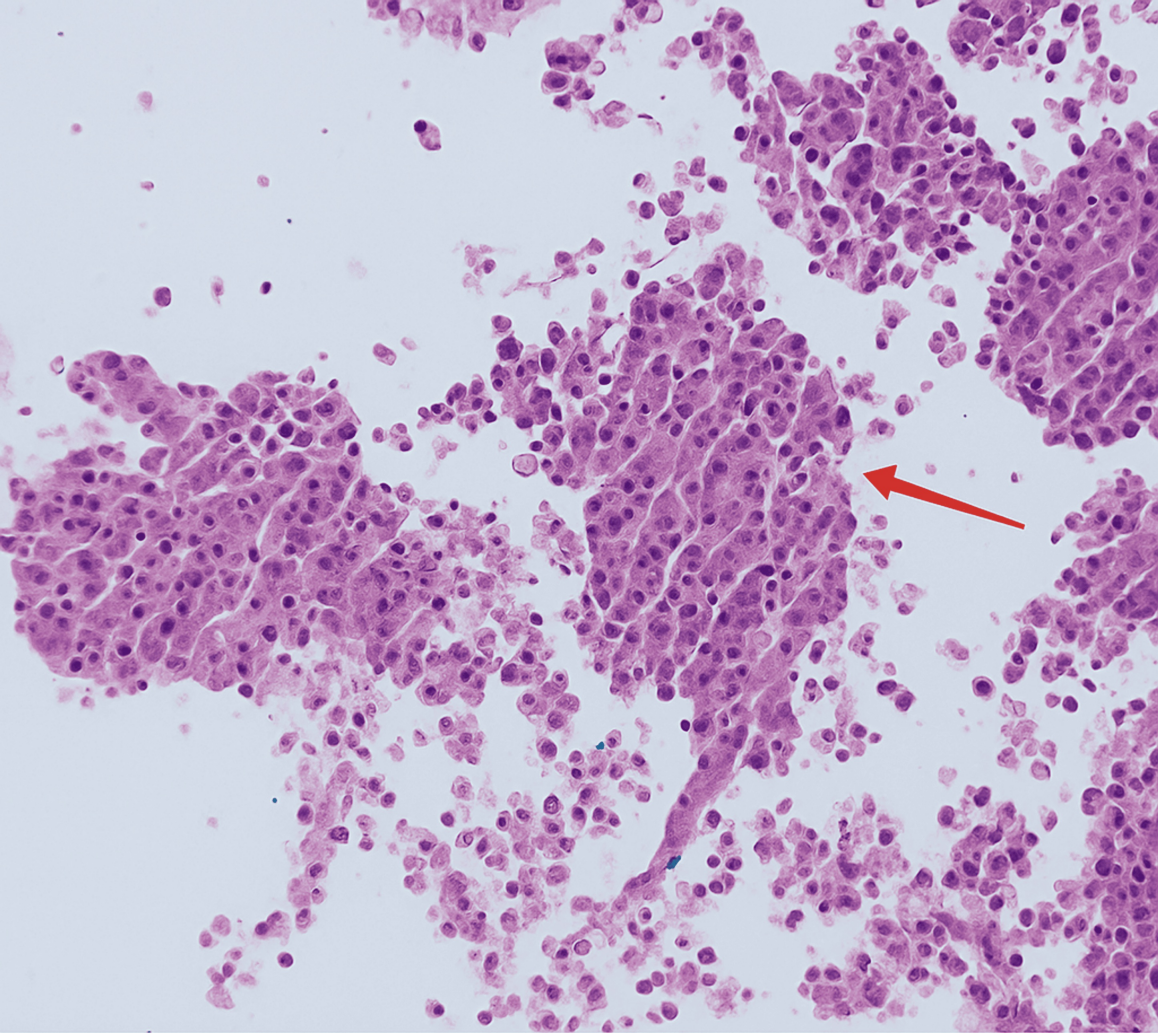
Immunohistochemical study result performed on left interlobar lymph node Extensive necrosis was seen in Station 11L (red arrow).

**Figure 6:**
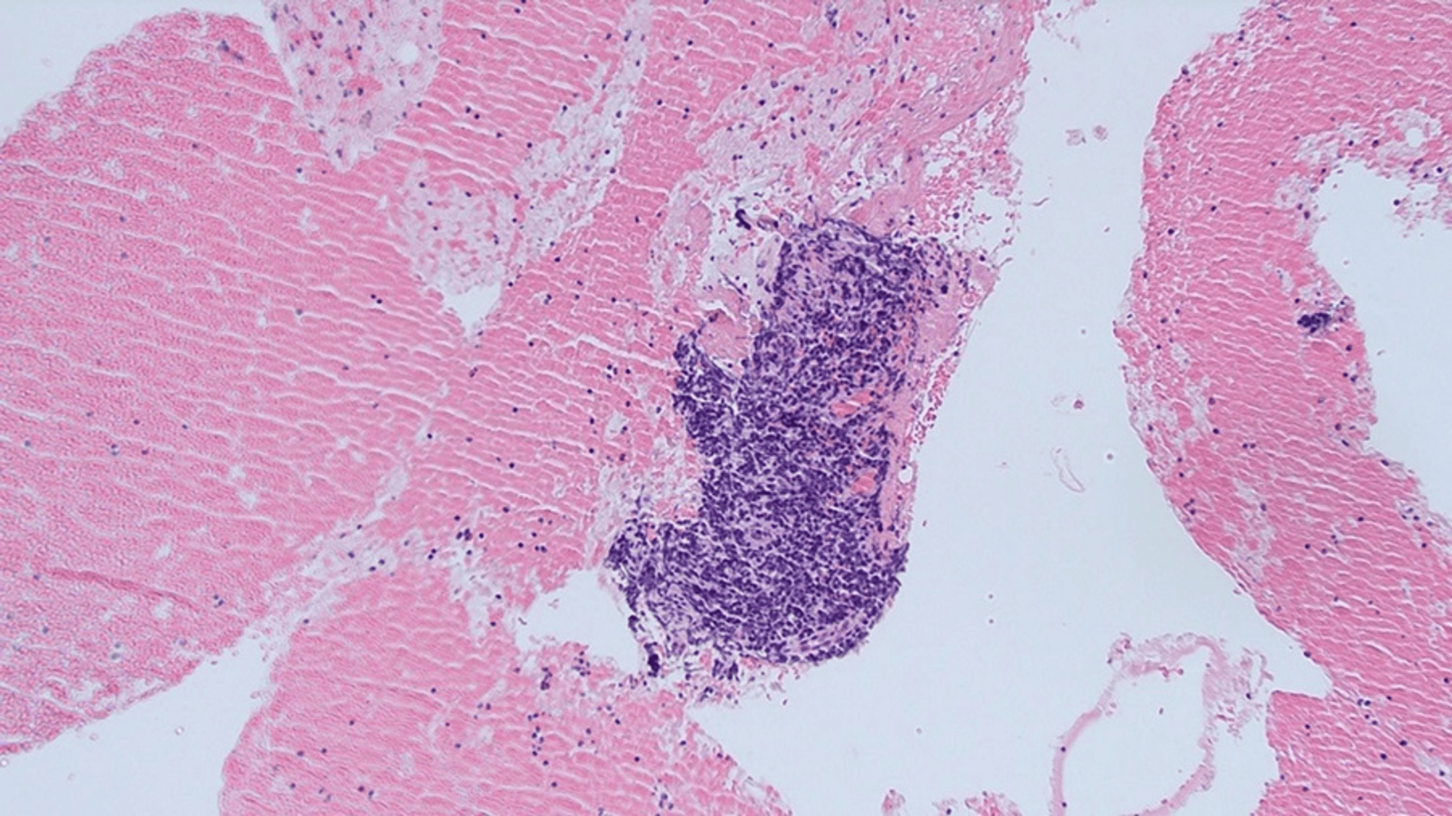
No evidence of malignancy in station 7

A previously healthy 33-year-old male developed cough and fatigue in December 2022. CT imaging on March 31, 2023, revealed a 9.1 x 9.7 cm left upper lobe (LUL) lung mass with small mediastinal lymphadenopathy. A subsequent PET/CT scan on April 18, 2023, showed the LUL mass with an SUV of 32 and a mediastinal node with an SUV of 3.8 (Figures 1, 2). A core needle biopsy of the LUL mass indicated a poorly differentiated malignant neoplasm suggestive of non-small cell lung cancer (NSCLC) with focal TTF-1 positivity (Figure 3). Molecular testing revealed an EML4-ALK rearrangement and high PD-L1 expression (99%). An MRI of the brain on April 25, 2023, showed no evidence of metastases (Figure 4). On May 9, 2023, endobronchial ultrasound with biopsy revealed extensive necrosis in station 11L (Figure 5) with no evidence of malignancy in station 7 or subcarinal lymph nodes (Figure 6).

The patient was discussed in a tumor board, and it was determined that maximum response to neoadjuvant therapy would be necessary if he were to proceed to resection and avoid a pneumonectomy. Neoadjuvant chemoimmunotherapy with carboplatin, paclitaxel, and nivolumab was initiated on May 12, 2023. However, the patient experienced a hypersensitivity reaction to nivolumab [4] and later developed anaphylaxis to paclitaxel, requiring brief hospitalization. A CT chest on June 3, 2023, showed a fair response. Given the patient's molecular profile, a targeted approach for the known ALK fusion protein was considered most beneficial, based on exceptional response rates from a randomized open-label phase III trial that demonstrated a 12-month event-free survival rate of 72.5% (95% CI, 64.6 to 80.4) in the alectinib group and 44.1% (95% CI, 34.5 to 53.6) in the crizotinib group [5].

**Figure 7:**
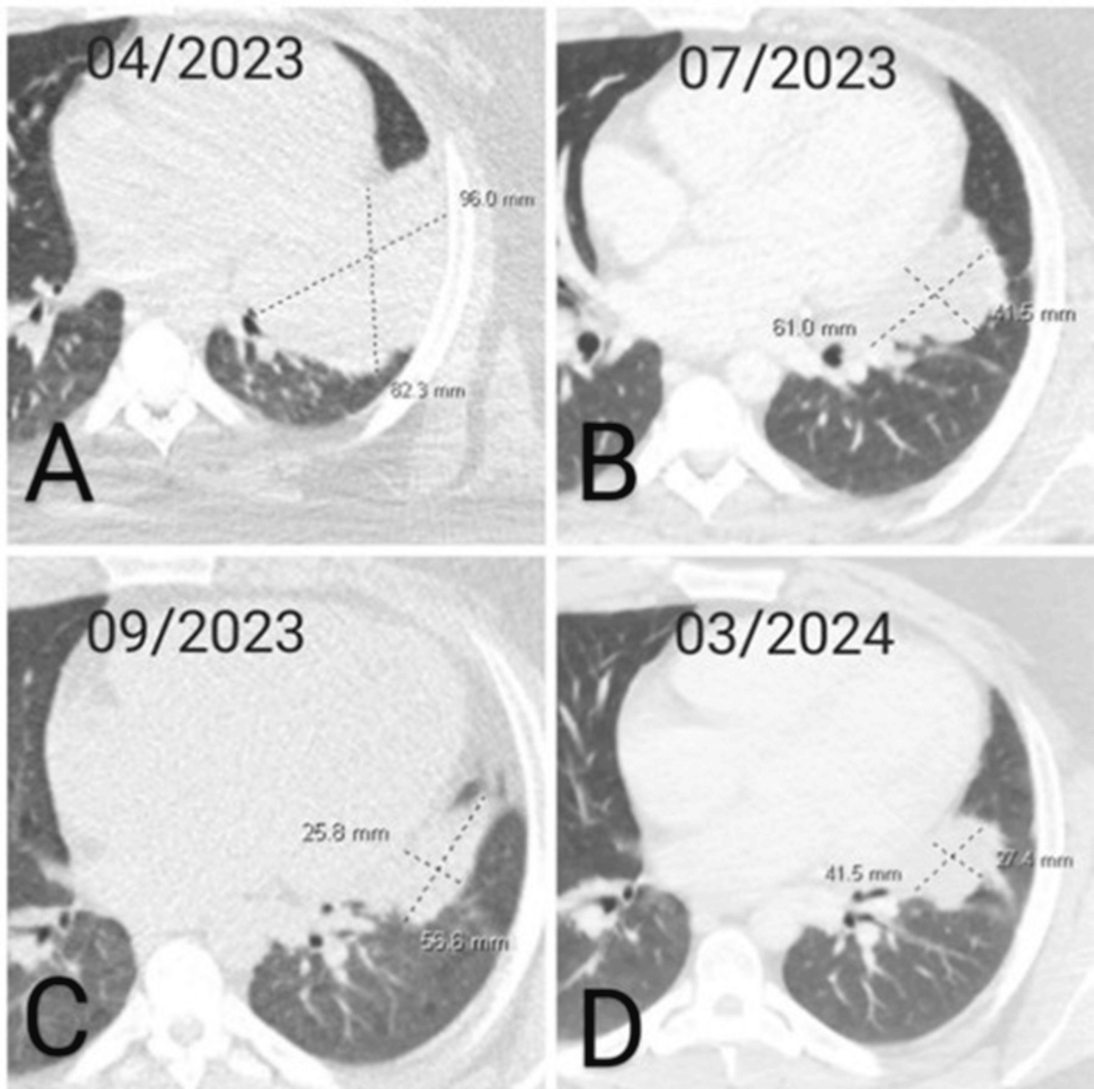
CT chest evolution from April 2023 to March 2024

On June 23, 2023, the patient initiated alectinib at 600 mg twice daily, with informed consent. He tolerated the therapy well, except for an impressive photosensitivity rash, a known adverse effect of the drug, which resolved after holding therapy for one week and administering prednisone. Subsequent CT imaging on July 14, 2023, and September 21, 2023, showed an impressive response to treatment, with no evidence of disease progression at other sites, as seen in Figures 7A-7D. In April 2024, the patient underwent a left upper lobectomy, left lower lobe wedge resection, and mediastinal lymph node dissection, with pathology showing a complete pathological response and no evidence of any residual tumor. Subsequently, the patient was started on adjuvant alectinib [6], and his circulating tumor DNA (Signatera MRD) was negative one month postoperatively.

## Discussion

Improved understanding of the molecular drivers of cancer and the subsequent development of targeted therapies have dramatically improved the outlook for many patients. The patient’s diagnosis of ALK-mutant NSCLC prompted the consideration of ALK inhibitor therapy. Since the PROFILE 1014 study established crizotinib as a more effective treatment option compared to chemotherapy in terms of both survival and quality of life, ALK inhibitors have gained traction [7]. Notably, the introduction of second-generation drugs in this class, such as alectinib and brigatinib, along with third-generation lorlatinib, has expanded treatment options for ALK-mutant lung cancer, surpassing the previous use of crizotinib [8].

Alectinib, a central nervous system (CNS) penetrant, was recognized through the ALEX [4] and J-ALEX trials [5] to be better suited for the treatment of ALK-positive NSCLC due to its propensity for CNS metastases. Moreover, the trials reported decreased frequency of adverse events and subsequent treatment adjustment. Through the ALTA trials [3], brigatinib was also deemed superior to crizotinib, with the reported 12-month progression-free survival rate of 67% as compared to 43% with crizotinib. Similarly, lorlatinib, which has been shown to overcome several ALK resistance mutations, demonstrated potent intracranial activity in the CROWN trials [6]. However, it was noted that lorlatinib affected weight, lipid panel, mood, memory, and cognition more frequently [9].

Alectinib was chosen in this case to minimize the CNS adverse effects associated with lorlatinib. Moreover, the side effect profile seen with crizotinib, such as nausea (48%), vomiting (38%), diarrhea (45%), elevated AST (30%) and ALT (25%) peripheral edema (28%), was greatly reduced with alectinib (14%, 7%, 12%, 15%, 14%, and 17%, respectively). Serious adverse events were reported to be 28% with alectinib as compared to 29% with crizotinib, including two deaths among patients treated with crizotinib. Given the patient’s adverse reaction to chemoimmunotherapy and the limited efficacy of immunotherapy in ALK-related lung cancer, alectinib was a rational choice. It is worth noting that, as of this report, alectinib is only FDA-approved for metastatic disease, as indicated by the ALEX study [4]. There are ongoing trials exploring its adjuvant use, such as the ALINA trial and isolated case reports [10]. The ALINA trial reported a two-year survival rate of 93.8% (n=130) among patients with resected Stage II or IIIa ALK-positive NSCLC [10]. This case report underscores the importance of personalized treatment approaches in advanced NSCLC, especially in the context of driver mutations such as ALK rearrangements.

## Conclusions

This case demonstrates the potential efficacy of neoadjuvant alectinib in managing stage III ALK-mutant lung adenocarcinoma, particularly when traditional chemoimmunotherapy is not well-tolerated. The significant tumor reduction and complete pathological response observed in this patient align with findings from the ALEX trial, which showed alectinib's superiority over crizotinib in terms of progression-free survival (34.8 months vs. 10.9 months, respectively) and reduced CNS metastases (12% vs. 45%, respectively). Furthermore, the ALINA trial reported a two-year disease-free survival rate of 93.8% in patients receiving adjuvant alectinib post-resection. These outcomes highlight the promise of targeted therapy in the neoadjuvant setting and reinforce the importance of personalized treatment strategies in advanced NSCLC. In patients with resected ALK-positive disease, follow-up care may include adjuvant ALK inhibitor therapy alongside routine imaging and molecular monitoring to detect recurrence or resistance early.
